# Respiratory support withdrawal in intensive care units: families, physicians and nurses views on two hypothetical clinical scenarios

**DOI:** 10.1186/cc9390

**Published:** 2010-12-29

**Authors:** Renata RL Fumis, Daniel Deheinzelin

**Affiliations:** 1 Unidade de Terapia Intensiva, Centro de Tratamento e Pesquisa Hospital AC Camargo, Rua Prof. Antônio Prudente, 211 - São Paulo, SP, Brazil CEP 01509-900; 2 Current address: Núcleo Avançado de Tórax, Hospital Sírio libanês, São Paulo, SP, Brazil

## Abstract

**Introduction:**

Evidence suggests that dying patients' physical and emotional suffering is inadequately treated in intensive care units. Although there are recommendations regarding decisions to forgo life-sustaining therapy, deciding on withdrawal of life support is difficult, and it is also difficult to decide who should participate in this decision.

**Methods:**

We distributed a self-administered questionnaire in 13 adult intensive care units (ICUs) assessing the attitudes of physicians and nurses regarding end-of-life decisions. Family members from a medical-surgical ICU in a tertiary cancer hospital were also invited to participate. Questions were related to two hypothetical clinical scenarios, one with a competent patient and the other with an incompetent patient, asking whether the ventilator treatment should be withdrawn and about who should make this decision.

**Results:**

Physicians (155) and nurses (204) of 12 ICUs agreed to take part in this study, along with 300 family members. The vast majority of families (78.6%), physicians (74.8%) and nurses (75%) want to discuss end-of-life decisions with competent patients. Most of the physicians and nurses desire family involvement in end-of-life decisions. Physicians are more likely to propose withdrawal of the ventilator with competent patients than with incompetent patients (74.8% × 60.7%, *P *= 0.028). When the patient was incompetent, physicians (34.8%) were significantly less prone than nurses (23.0%) and families (14.7%) to propose decisions regarding withdrawal of the ventilator support (*P *< 0.001).

**Conclusions:**

Physicians, nurses and families recommended limiting life-support therapy with terminally ill patients and favored family participation. In decisions concerning an incompetent patient, physicians were more likely to maintain the therapy.

## Introduction

While sophisticated technological support has allowed ICU (Intensive Care Unit) patients to survive longer, there is a widespread perception that intensive medical care at the end of life frequently represents excessive, inappropriate use of technology [1,2]. Recommendations on end-of-life and the potential conflicts about it, guidelines and consensus conferences are now available [3-6]. However, there are divergences of patients' and doctors' preferences regarding life support in such situations within countries and among different cultures and religions [7,8].

Throughout North America and Europe, between 40% and 90% of deaths in intensive care are preceded by the decision to withdraw or withhold life support [9]. Decisions to forgo life-sustaining therapy are commonly made worldwide and their frequency is increasing: in five years, the proportion of ICU deaths where such decisions were taken went from 51% to 90% [10]. Advanced care planning and effective ongoing communication among clinicians, patients and families are essential to improve end-of-life decision-making and reduce the frequency of a mechanically supported, painful and prolonged process of dying [11]. The decision to forego treatment is generally made by the medical team [12,13]. Although the participation of nursing staff in ethical decisions is recommended [6], the involvement of nurses was shown to vary from 16% (in a Canadian study) to almost 96% (in the USA) [2].

Family members of patients in the ICU are usually under severe stress [14,15] and often misunderstand the prognosis of the patient for whom they are making decisions [16]. In addition, families' dissatisfaction was associated with situations where disagreement between the physicians' and the families' perspective of prognosis occurred [17]. Nurse-physician disagreement regarding care in the ICU is common, especially for patients requiring treatment-limitation decisions. Several investigators pointed out the differences in professional values of nurses and physicians related to the dying process [18,19]. According to the patient's condition and prognosis, the decision to withdrawn life support gets more difficult [11] and there is little consensus about who should make it [12]. Conflicts at a patient end-of-life were associated with increased family and staff stress [12,20,21]. Nurse-physician communication is strongly related not only to better end-of-life care but also to the nurses' and physicians' job satisfaction [22].

In Sweden we used two clinical scenarios to examine the attitudes of the general public, nurses and intensive care physicians regarding who should make the decision on withdrawal of life support. It was discovered that, the general public favors more patient and family influence as compared with physicians' and nurses' (50%, 8%, 31%, respectively) [12]. There are indeed considerable differences in how physicians and the general public reason in critical care situations [23].

The objective of this study was to examine the views of the families, physicians and nurses in Brazilian Intensive Care Units regarding end-of-life decisions, involving a conscious and an unconscious patient.

## Materials and methods

ICUs were selected based on the following criteria: adult ICU, having more than six beds and more than two attending physicians daily. An invitation to participate was sent to the directors of 13 ICUs from Sao Paulo centre tertiary hospitals.

In order to obtain the opinions of ICU physicians and nurses, a questionnaire was sent to all possible nurses (215) and physicians (176) in the participating units. Data collected from all physicians and nurses were gender, age, religion, years of professional activity, years of ICU experience and characteristics of ICUs: type of ICU, type of hospital and number of beds.

Family members of consecutive cancer patients who stayed in the Hospital do Cancer ICU for more than 72 hours were also included. One family member per patient, defined as spouse, child, parent or sibling, was interviewed. Data collected from all families were gender, age, marital status, level of education, religion, relationship with the patient, previous experience with the ICU and their view of the prognosis. We also collected the physicians' views regarding patients' prognosis and final outcomes in the ICU. Families and physicians in charge were asked at the moment of the interview whether they expected the patient to survive (not severe) or not (severe). This surmise was compared with the final ICU outcome, generating a dichotomous variable referred to as a right or wrong prognosis. The non-concordance regarding prognosis was defined when the physician and the families' perspective of prognosis disagree.

To survey the attitudes regarding withdrawal of life-support the questionnaire developed by Sjökvist *et al. *(1999) was used [12]. The questionnaire consisted of two clinical scenarios (one with a conscious and competent patient with severe cancer and the other with an unconscious and incompetent patient that suffered head injuries in a serious accident and one month later was still unconscious) asking if the physician should raise the question of continued ventilator treatment and who should decide whether the ventilator treatment should be discontinued (See Appendix section in Additional file 1).

Informed consent to participate was given by all patients, physicians and nurses using the standardized hospital consent form including consent to publish.

The study was approved by Hospital do Cancer as well as by four participant hospitals ethics committees. The questionnaire was translated into Portuguese and back translated in order to be applied [16].

### Statistical analysis

For analysis purposes, continuous data were categorized according to the median. Contingency tables were constructed and analyzed with Chi-Square. A *P *< 0.05 was considered statistically significant. The SPSS 11.1 (SPSS, Chicago, IL, USA) was used for calculations. For analysis, an affirmative answer was considered whether the respondent marked each of the following answers "yes, with the patient only", "yes, with the family only, "yes, with both the patient and the family" in the first question of the first scenario. For the second question, regarding who should decide, answers were grouped as follows: "patient and/or family with the physician" or "patient and/or family without the physician" and "the physician only" (Tables 1 and 2). Stepwise logistic regression was used to better adjust for confounding variables of decisions to withdrawal life support.

**Table 1:** Differences between physicians' and nurses' opinion about discussing withdrawal of continued ventilation with the family

	Physicians (%) N = 155			Nurses (%) N = 204		
	Yes	No	Uncertain	Yes	No	Uncertain
Competent patient	116 (74.8)	35(22.6)	4(2.6)	153(75.0)	46(22.5)	5(2.4)
Incompetent patient	94 (60.7)	54(34.8)	7(4.5)	151(74.0)	47(23.0)	6(3.0)

Chi-Square = 7.27, *P *= 0.026 for the differences between the physicians and nurses when the scenario is with incompetent patient. Chi-Square = 7.18, *P *= 0.028 for the differences of the physicians' opinions when change the scenario.

**Table 2:** Scenarios with competent patient and with incompetent patient: Who should decide about continued ventilator treatment?

	Physicians (%) N = 155			Nurses (%) N = 204		
	Patient and/or family without the physician	Patient and/or family together with the physician	The physician only	Patient and/or family without the physician	Patient and/or family together with the physician	The physician only
Competent patient	32 (20.6)	111 (71.6)	8 (5.2)	80 (39.2)	109 (53.4)	10 (4.9)
Incompetent patient	11 (7.1)	119 (76.8)	16 (10.3)	28 (13.7)	160 (78.4)	10 (4.9)

Chi-Square = 14.5, *P *= 0.001 for the differences between the physicians and nurses with competent patient. Chi-Square = 7.12, *P *= 0.028 for incompetent patient. Chi-Square = 13.1, *P *= 0.001 for the differences of physicians' opinions when the scenario changes. Chi-Square = 34.7, *P *< 0.0001 for the differences of the nurses' opinions when the scenario changes.

## Results

Out of 13 Hospitals from Sao Paulo centre approached to participate in this study, 12 (92.3%) agreed to do it. Within these 12 hospitals, 155 (88%) of potentially eligible 176 ICU physicians and 204 (94.5%) of 215 ICU nurses participated.

The median of ICU beds was 24 (range 9 to 40). Seven hospitals were university affiliated (58.3%) and state hospitals comprised 25% of total. All participating ICUs were mixed medical/surgical and one was exclusive for neurological patients.

Table 3 shows the distribution of characteristics of the intensivists, nurses and families. All 155 physicians answering the questionnaire were intensive-care specialists. All families were proceeding from a medical-surgical ICU in a Tertiary Cancer Hospital. A total of 443 eligible patients were identified during the study period. Of these, 300 families were interviewed. The 143 remaining did not participate for different reasons: 28 did not meet the inclusion criteria; 14 were not contacted during the visiting periods; 20 alleged they had no time to participate; 26 felt unable to participate; 39 did not attend our invitation and, finally, 16 patients never received visits. Families were interviewed in a median of four (three to five) days after patient entrance. We found that a large percentage of family members (29%) did not have previous experience with the ICU. Failure to comprehend the prognosis was noted in 23.7% of the family members. We also identified that 16.3% families did not agree with the physicians' views about the final outcome in ICU.

**Table 3:** Demographic description of physicians, nurses and family members interviewed

*Related intensivists (N = 155)*	
Female N (%)	121 (78)
Male	34 (22)
Age (Median)	41(28 to 70)
Time since Graduation	17.00 (5 to 43)
ICU experience (Median)	14.00 (<1 to 37)
Catholic Religion N(%)	92 (59.3)
***Related nurses (N = 204)***	
Female N (%)	185 (90.7)
Male	19 (9.3)
Age	33 (22 to 61)
Time since Graduation	8 (1 to 33)
ICU experience	6 (<1 to 32)
Catholic Religion N (%)	111 (54.4)
***Related family members (N = 300)***	
Female	195 (65)
Male	105 (35)
Age	45 (20 to 80)
Marital status (married)	207 (69)
Catholic religion	175 (58.3)
Level of education	
Elementary school	38 (12.7)
High school	94 (31.3)
College education	168 (56)
Relationship offspring N (%)	168 (56)
spouses N (%)	83 (27.7)
Previous knowledge of ICU N (%)	213 (71)

Table 4 shows the differences between the three groups in both scenarios. Regarding decision-making when the patient is competent, we observed that the majority of families (78.6%), physicians (74.8%) and nurses (75%) favored the physician raising the question about withdrawal of ventilator support. Most of families (66.3%), physicians (71.6%) and nurses (53.4%) wanted to share the decision responsibility together with the patient and/or family. Still in this scenario, only 5.2% of physicians answered that they alone should be the ones to make the decision, a view held by 4.9% of the nurses and by 4.3% of families. However, the combination of the patient and/or the family without the physician as decision-makers were significantly more supported by nurses (39.2%) as compared to families (28%) and to physicians (20.6%) (*P *< 0.001).

**Table 4:** Differences according to scenarios for decisions about continued ventilator and for who should decide

Should physicians raise the question about withdrawal the ventilator? Scenario with competent patient	Family N (%)	Physician N (%)	Nurse N (%)	*P*-value
Yes	236 (78,6)	116 (74,8)	153 (75,0)	0.628
No	59 (19,7)	35 (22,6)	46 (22,5)	
Scenario with incompetent patient				
Yes	244 (81,3)	94 (60,7)	151 (74,0)	<0.0001
No	44 (14,7)	54 (34,8)	47 (23,0)	
Scenario with competent patient				<0.001
Who should decide?				
Patient and/or family without physician	84 (28,0)	32 (20,6)	80 (39,2)	
Patient and/or family together with physician	199 (66,3)	111 (71,6)	109 (53,4)	
Physician only	13 (4.3)	8 (5.2)	10 (4.9)	
Patient only	22 (7.3)	21 (13.5)	35 (17.1)	
Family only	25 (8.3)	1 (0.6)	6 (3.0)	
Scenario with incompetent patient Who should decide?				0.077
Family only	42 (14,0)	11 (7,1)	28 (13,7)	
Family and physician together	236 (78,7)	119 (76,8)	160 (78,4)	
Physician only	19 (6.3)	16 (10.3)	10 (4.9)	

When the patient was incompetent, physicians (34.8%) were significantly more prone than nurses (23.0%) and families (14.7%) (*P *= 0.026) to reject decisions regarding withdrawal of the ventilator support. We observed that the minority of physicians (10.3%), families (6.3%) and nurses (4.9%) suggested that the physician should be the sole decision-maker. The majority of families (78.7%), physicians (76.8%) and nurses (78.4%) pointed out that the family and the physician should make the decision together.

Tables 1 and 2 emphasize the differences between physicians and nurses according to the change of scenarios. Physicians are more likely to propose withdrawal of the ventilator and share decisions with competent patients as compared to incompetent patients (74.8% vs 60.7%, *P *= 0.028). We observed that nurses' opinions regarding who should decide were very different depending on whether the patient was competent or incompetent (*P *< 0.0001).

When the patient is competent, we observed that patients for whom family decisions were made to withdraw life-support therapies had poorer prognosis (89.3% vs 75%, *P *= 0.003) and prolonged mechanical ventilation needs (84.4% vs 74.8%, *P *= 0.041). We also found that families with higher education were more likely to decide for withdrawal (84.2% vs 74.6%, *P *= 0.040). Physicians with no Catholic affiliation were more willing to withdraw life sustaining therapies (86.9% vs 70.3%, *P *= 0.013). We found that in cases with incompetent patients, the child as compared with others relatives (90% vs 78%, *P *= 0.006) and families with higher education (88.7% vs 79.8%, *P *= 0.038) were more likely to withdraw life sustaining therapies. Stepwise logistic regression disclosed that physician's with no Catholic affiliations were more likely to recommend withdrawal of life support (OR 2.74, CI 1.15 to 6.54). Regarding families, we found that a poor comprehension of prognosis (OR 2.42, CI 1.07 to 5.49), high level of education (OR 2.13, CI 1.15 to 3.85) and a child's condition (OR 2.63, CI 1.34 to 5.18) favored decisions to withdraw life support. We also found that for patients with severe a prognosis (OR 3.89, CI 1.81 to 8.34) and with metastasis (OR 2.32, CI 1.19 to 4.53), family members were more likely to decide for withdrawal of life support (Table 5).

**Table 5:** Multivariate analysis of predictors for decision to withdrawal life support using stepwise logistic regression

Scenarios	Category	OR (95% CI) crude	*P*-value	OR (95% CI) multivariate	*P*-value
**Scenario with competent patient**					
	***Related to the physicians***				
	No catholic affiliations	2.74 (1.15 to 6.54)	0.023	2.74 (1.15 to 6.54)	0.023
	***Related to the family***				
	Poor Comprehension of prognosis	2.12 (0.95 to 4.74)	0.066	2.42 (1.07 to 5.49)	0.034
	High level of education	1.82 (1.02 to 3.23)	0.042	2.13(1.15 to 3.85)	0.015
	***Related to the patients***				
	Prolonged MV*	1.82 (1.02 to 3.24)	0.042	-	-
	Severe prognosis	2.79 (1.38 to 5.64)	0.004	3.89 (1.81 to 8.34)	<0.001
	Metastasis	1,69 (0,91 to 3,16)	0,099	2.32 (1.19 to 4,53)	0,014
**Scenario with incompetent patient**					
	***Related to the family***				
	High level of education	1.98 (1.03 to 3.80)	0.041	-	-
	Child	2.48(1.27 to 4.82)	0.007	2.63 (1.34 to 5.18)	0.005

* Mechanical ventilation

## Discussion

In this study conducted in Sao Paulo, the largest city of Brazil and of South America, we report physicians', nurses' and families' high rates of decisions to withdraw life support. Our findings agree with the attitudes of the Swedish population that acknowledged the right to refuse life-sustaining treatment, including life support [12]. However, we found that Brazilian physicians differ from the Swedish physicians surveyed by Sjokvist [12]. While in our study physicians emphasized shared decision making, Swedish physicians demonstrated a higher proportion of intention to be a sole-decision maker for physicians in the incompetent patient scenario. Concerning families' and nurses' opinions, we observed that they are in accord with the Swedish' public and nurses, who favor more patient and family influence in end-of-life decisions. Differently from the Swedish study [12], which addressed the general public, our families were of cancer patients. Although differences in acuity and understanding of prognosis may exist between those populations, it must be noted that cancer predicts limitation of therapy in a similar manner of other chronic conditions and, therefore, we do not believe in an unplanned bias [24,25].

We observed that some family members said that they were unable to participate and others did not attend our invitation. Although information on families who refused to participate was not gathered, we have previously observed that when the patient was too ill, families felt unable to participate [16]. Moreover, previous researchers have documented clinically significant psychological distress among advanced cancer patient caregivers and that maybe another explanation for non-participation [26]. Because most critically ill patients are unable to participate in end-of-life decisions, family members are generally asked to participate. Few surveys have explored the views of a close family member of seriously ill patients [1,9,27]. However, family participation rates in decision making vary across countries, due to both staff and family reasons [28]. Families in France, for instance, participated in decision making in 44% of the cases [2], contrasting to up to 80% participation in the US [10,29]. In Canada, surveys disclosed that 87% of the public favored the family as a decision-maker for an incompetent patient and 84% supported the right to withdraw life support from a comatose patient [30,31].

In a large multicenter study on the incidence of conflicts, the authors reported that decisions to forgo life support were routinely shared with family members in one-third of ICUs. However, conflicts on such decisions were perceived as "severe" and "dangerous" by up to 50% of the respondents and poor communication within the ICU was perceived as a major cause [20].

The major disagreement that we observed between nurses and physicians was about end-of-life decisions with an incompetent patient, which is important since that is the most common case where such decisions are needed [7]. In the incompetent patient scenario, a case of head injury, physicians feel less inclined to withdraw life support. We found that 81% of families and 74% of nurses wanted to discuss withdrawal against only 61% of physicians. Differently, incompetent patients are associated with more end-of-life decision-making [7] and we could observe that the neurological system failure is one of the reasons for withdraw life support [2,24,25]. Regarding how frequently trauma patients are removed from life support, such decision varies across trauma centers (0 to 16%) what points to the prognostic complexity of these situations [32]. In the competent patient scenario, ventilator assistance was due to severe cancer and pneumonia, a situation shown to be more frequent in patients with decisions to limit therapy [2]. Interestingly, some cultures diverge concerning the role of surrogates in the case of mentally incapacitated patients. North American relatives, by right, share the decisions with physicians. In Europe, guidelines agree that proxies, whose preferences are to be taken into account, should be informed, but do not have the right and the responsibility for the decision [33].

Nurses considered significantly more often that the patient and/or the family alone should make the decisions in a frequency similar to that reported in Sweden [12]. Although clinicians have the ultimate responsibility, they often fail to predict patient desires regarding end-of-life treatments [6]. Since nurses often have closer and prolonged contact with patients and their families, they may provide valuable insights into patient/family feelings and opinions [18] as well as favor the family and patient as decision-makers [19]. Nonetheless, critical care nurses expressed extreme frustration about their limited role in the management of patients at end of life [34]. Notwithstanding the above, the fact that physicians were older, had more ICU experience and were mostly male could also explain our results, which was not tested in the present study.

Religious affiliations usually influence physicians' attitudes toward withdrawal of life support [5,35]. Our data are in accordance with European studies that showed a similar willingness to discuss withholding of treatment and that such discussion occurred less often if the physician was Catholic [35]. Moreover, in Italy, a country of strong Catholic tradition, the proportion of physicians who admitted foregoing treatment was lower than in other Europe countries [13].

This study is limited in that the respondents reacted to hypothetical scenarios and how they really act is unknown. Studies have reported that Brazilian physicians are more prone to withhold treatments than to actively stop or withdraw life-sustain treatments [8,36]. The study was carried out in 12 hospitals from a single city (Sao Paulo) and it cannot be viewed as an audit of Brazilian ICU physicians' and nurses' opinions, despite it taking place in the main city of South America. Another limitation is that, even though nurses should participate in the process to limit care [6], their actual role was not assessed. Finally, families' interview was conducted in a single centre and therefore may have been influenced by local factors.

Studies found that patients would rather have their families and physicians jointly making end-of-life decisions [6]. However, most European physicians believe that withholding and withdrawing life support are predominantly biomedical and ethical issues and therefore they should make such decisions alone [12,13]. Furthermore, family members are not always willing to share the decision-making process [28]. Aware of such problems, the Brazilian Federal Council of Medicine issued a resolution, which is still in debate in the country, that reinforces the appropriate life support limitation measures to patients deemed as in an irreversible condition [37],. Nonetheless, the principle of respect for patient autonomy has come to dominate medical decision-making in the United States and other countries [38].

Although our findings seem to contradict the traditional view of Brazilian's physicians as having a paternalistic approach, we believe that this potential change reflects an increasing debate over appropriate terminal care over the last two decades [25,33]. Furthermore, end-of-life research has grown considerably in quality and quantity and provides insights into attitudes toward death in the intensive care unit and withdrawal of life support in particular [39]. In the USA, deaths preceded by decisions to forgo life-sustaining treatments increased from 51% in 1987 to 1988 to 90% in 1992 to 1993 [10]. In Canada, the rate of life-sustaining limitation range from 65% to 80%, and in Europe range from 23% to 86.5% [33]. Whereas in North America withdraw treatments appear to be a common way to limit care, in Europe physicians are uncomfortable with this, especially those with strong religious beliefs and those from the South [33]. Similarly, in Brazil, medical staffs still have some difficulty in assuming the life support limitation, which could be related to legal concerns [36], although a Brazilian study reported a progressive increment of Life Support Limitation (LSL) from 6% in 1988 to 36% in 2002 [40].

Family members of ICU patients disclose a high prevalence of anxiety and depression, particularly when facing poor prognosis [14,15]. Because of this, special attention on ICU physician accessibility and full information provided by the ICU staff are essential [14,17]. Furthermore, we found that poor comprehension of prognosis was associated with more willingness to withdraw life-support. Whether a better comprehension of prognosis would change such willingness is beyond the scope of the present study.

We have shown that families, physicians and nurses are willing to discuss end-of-life-decisions. End-of-life conferences with the family are fundamental [41], but better consensus between physicians and nurses, who disagreed in the present scenarios, must be reached in order to provide uniform information.

## Conclusions

The present study indicates that although the majority of physicians, nurses and family members agree that decisions to withdraw life support should be made, significant differences still exist, particularly regarding surrogate decisions for an incompetent patient. The majority of the physicians and nurses prefer family involvement in end-of-life decisions. In order to avoid unnecessary mismatched communication, physicians and nurses should have a better consensus about end-of-life decision-making.

## Key messages

• Physicians and nurses emphasized that decisions should be shared and favored family participation.

• Physicians are more likely to reject decisions regarding withdrawal of the ventilator support of an incompetent patient.

• The major disagreements between physicians and nurses occurred when a decision concerned an incompetent patient.

• Physicians should pay special attention to poor prognosis, since in such cases family members are inclined to decide for withdrawal of life support.

• Physicians and nurses should have a consensual view before approaching the family for end-of-life conferences.

## Abbreviations

MV: mechanical ventilation.

## Competing interests

The authors declare that they have no competing interests.

## Authors' contributions

Both authors contributed equally to the manuscript.

## Supplementary Material

Additional file 1 **The Appendix**. The questionnaire.doc. Click here for file
