# Supplementary material for: Respiratory support withdrawal in intensive care units: families, physicians and nurses views on two hypothetical clinical scenarios
Source: Crit Care. 2010 Dec 29;14(6):R235. doi: 10.1186/cc9390 (PMC3220008; doi:10.1186/cc9390)
Supplement: Additional file 1 — The Appendix. The questionnaire.doc. [file cc9390-S1.DOC]

Appendix - The Questionnaire

| Scenario with conscious and competent patient |
| --- |
| A 60-year-old married woman with severe cancer and pneumonia needs the assistance of a ventilator in order to breathe. The woman will die within 24 h if the ventilator is withdrawn. The woman’s physician is completely convinced that she will die within a period of 1 month regardless of what treatment she receives. The woman is exhausted by her severe disease, but fully conscious and able to express her wishes. The physician is considering withdrawing the ventilator and allowing her to die, so she will no longer have to suffer.  Do you believe that the physician should raise the question of continued ventilator treatment with the patient and family, i.e. the patient’s husband and children? |
| 1. Yes, with the patient only 2. yes, with the family only 3. yes, with both the patient and the family 4. No, the physician should not raise the question with the patient and the family 5. Uncertain |
| Assuming that the physician has brought up the question of ventilator treatment for discussion, who do you believe should decide whether or not the ventilator treatment should be continued? |
| 1. The patient only 2. The family, i.e. husband and children, only 3. The physician only 4. The patient and the family together 5. The patient and the physician together 6. The family and the physician together 7. The patient, the family and the physician together 8. Uncertain |
| Scenario with unconscious and incompetent patient |
| A 60-year-old married woman was in a serious accident in which she suffered head injuries. One month later she is still unconscious and needs the assistance of a ventilator in order to breathe. The woman will die within 24 h if the ventilator is withdrawn. The physician is completely convinced that she will not wake up, although she might live for a while if the ventilator is kept in place. The physician is considering withdrawing the ventilator treatment and allowing her to die.  Do you believe that the physician should raise the question of continued ventilator treatment with the family, i.e. the patient’s husband and children? |
| 1. Yes 2. No 3. Uncertain |
| Assuming that the physician has brought up the question of ventilator treatment for discussion with the family, who do you believe should decide whether or not the ventilator treatment should be continued? |
| 1. The family only 2. The physician only 3. The family and the physician together 4. Uncertain |
